# Recent Developments in Nanofiber Fabrication and Modification for Bone Tissue Engineering

**DOI:** 10.3390/ijms21010099

**Published:** 2019-12-21

**Authors:** Nopphadol Udomluck, Won-Gun Koh, Dong-Jin Lim, Hansoo Park

**Affiliations:** 1 School of Integrative Engineering, College of Engineering, Chung-Ang University, 84 Heukseok-ro, Dongjak-gu, Seoul 06974, Korea; n.udomluck@gmail.com; 2 Department of Chemical and Biomolecular Engineering, YONSEI University, 50 Yonsei-ro, Seodaemun-gu, Seoul 03722, Korea; wongun@yonsei.ac.kr; 3 Otolaryngology Head & Neck Surgery, University of Alabama at Birmingham, Birmingham, AL 35233, USA

**Keywords:** electrospinning, melt-electrospinning, biomolecule delivery, 3-dimensional nanofiber, bone engineering

## Abstract

Bone tissue engineering is an alternative therapeutic intervention to repair or regenerate lost bone. This technique requires three essential components: stem cells that can differentiate into bone cells, growth factors that stimulate cell behavior for bone formation, and scaffolds that mimic the extracellular matrix. Among the various kinds of scaffolds, highly porous nanofibrous scaffolds are a potential candidate for supporting cell functions, such as adhesion, delivering growth factors, and forming new tissue. Various fabricating techniques for nanofibrous scaffolds have been investigated, including electrospinning, multi-axial electrospinning, and melt writing electrospinning. Although electrospun fiber fabrication has been possible for a decade, these fibers have gained attention in tissue regeneration owing to the possibility of further modifications of their chemical, biological, and mechanical properties. Recent reports suggest that post-modification after spinning make it possible to modify a nanofiber’s chemical and physical characteristics for regenerating specific target tissues. The objectives of this review are to describe the details of recently developed fabrication and post-modification techniques and discuss the advanced applications and impact of the integrated system of nanofiber-based scaffolds in the field of bone tissue engineering. This review highlights the importance of nanofibrous scaffolds for bone tissue engineering.

## 1. Introduction 

**Figure 1:**
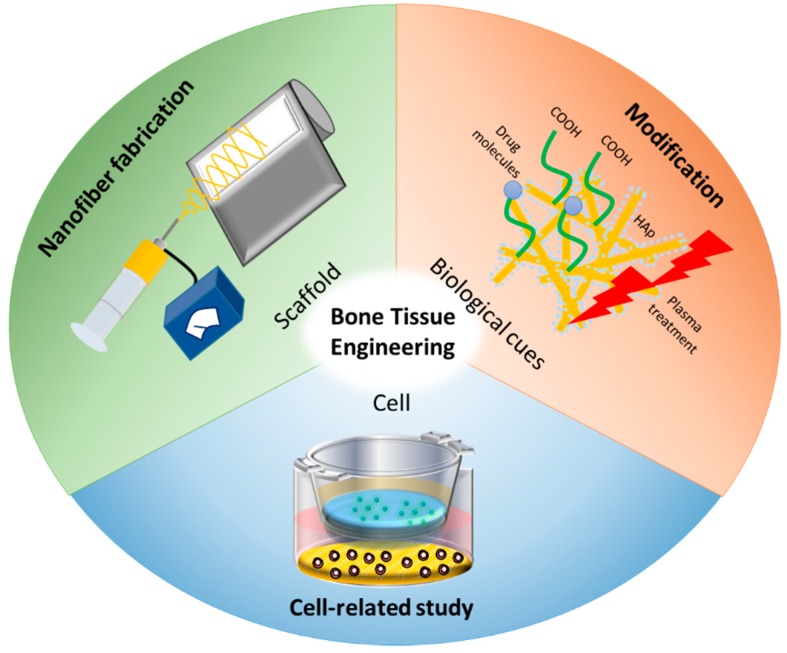
Schematic diagram of bone tissue engineering strategies.

Bone tissue engineering is an alternative therapeutic treatment for damaged bones. Compared to traditional tissue transplantation, including autografts and allografts, bone tissue engineering techniques eliminate the problems of donor insufficiency, supply limitations, and immune rejection [1]. Tissue engineering strategies make use of scaffolds in combination with biological supplements and cells (Figure 1). The critical challenge in bone tissue engineering is the construction of a bio-artificial bone implant, a scaffold imitating the extracellular matrix (ECM) with osteoconductive function, that results in the restoration of injured or diseased bones [2]. One essential bone tissue scaffold property is the overall porosity that allows the integration of large numbers of osteogenic cells to form tissues [3]. In general, it has been confirmed that a structure with high porosity, interconnected pores, and a large surface area exhibits positive results regarding tissue in-growth [4]. Among the many types of scaffolds, nanofibrous scaffolds have gained attention due to their suitable properties for bone tissue engineering [5,6].

Nanofibrous scaffolds represent a potential platform for use in bone tissue engineering, as they allow promising fabrication with physical properties that mimic the ECM [7]. Within the last decade, the electrospinning method has become a well-known technique for preparing polymeric nanofibers and nonwoven mats with diameters of a few nanometers [8,9]. Electrospun fibrous scaffolds can be fabricated using a variety of developed tools for electrospinning, including co-axial spinnerets, heating systems, triaxials, needles, patterned collectors, and negatively charged electrodes [10]. To increase the potential of electrospun fibrous scaffolds for application, many studies have extensively focused on not only the fabrication but also the enhanced functional performance of the nanofibers [11,12,13,14]. For bone restoration, it is critical to activate the self-regeneration mechanism that promotes new bone formation while also ensuring the creation of a new vascular supply [15]. Functionalized or reinforced nanofibrous scaffolds may be able to create a hospitable ECM-mimicking environment that helps migration and proliferation of cells, since the ECM in particular has been recognized as an active depot of growth factors that regulate growth factor activity [16]. 

Growth factors are important for bone formation. The most well-studied bone growth factors are platelet-derived growth factor (PDGF), insulin-like growth factor (IGF), transforming growth factor beta (TGF-β), bone morphogenetic protein (BMP), and basic and acidic fibroblast growth factor (bFGF and aFGF, respectively) [17,18]. These growth factors affect signaling, proliferation, and differentiation of bone cells [18]. Growth factors also participate in angiogenesis, vascular maturation, and subsequent maintenance of the established vasculature [19]. In addition, the interplay of these three processes affects successful bone regeneration. The ECM can act as a reserve for growth factors and is involved in the dynamic interaction between cells and growth factors [20]. Growth factors in bone formation stimulate the migration and differentiation of osteoprogenitor cells through sequential expression of angiogenic, inflammatory, and bone growth [21]. Bound growth factors in the ECM are actively capable of providing cues that initiate bone formation [16]. For example, recombinant human bone morphogenetic protein 7 (rhBMP-7) was successfully used to demonstrate the bone formation of critical-sized defects (larger than 10 mm) in sheep when formulated with autologous bone marrow-derived mesenchymal stem cells (MSCs) and a biodegradable composite scaffold made of medical grade polycaprolactone (PCL) and tricalcium phosphate [22]. Similarly, BMP-2 is used for bone formation. These two bone morphogenetic proteins are the only growth factors for bone regeneration approved by the FDA (The Food and Drug Administration) [23]. Furthermore, FGF-2 stimulates the growth of osteoprogenitor cells in the bone formation stage [24]. To deliver growth factors in each stage of bone healing without compromising essential features of an ideal scaffold for bone regeneration, a well-interconnected biocompatible material should be utilized. Providing a well-tailored bone scaffold with controlled delivery of growth factors can offer successful bone regeneration. Moreover, providing efficacious and cost-effective growth factor delivery is crucial for the clinical success of bone regeneration [25].

To mimic the nature of the ECM as a reservoir of bone growth factors, a facile and flexible technique to fabricate nanofibers, referred to as electrospinning, has been widely studied [26]. Scaffolds made by electrospinning have great potential to create a favorable microenvironment that supports bone formation [27,28]. Not only does this technique create nanofibrous scaffolds for bone tissue engineering, it is also used for sustained release of growth factors [29]. Electrospinning enables the fabrication of fibrous scaffolds composed of different materials with excellent spatial interconnectivity. As in nature, the protein fibers in the ECM are in the form of fibrillar collagen and elastin. Electrospinning represents an attractive technique that can be used to create a synthetic ECM. The hierarchical structure of the ECM can be easily mimicked by electrospun nanofibers such that bone-related cells are able to initiate the process of bone regeneration [30]. Moreover, the versatility of fabricating electrospun nanofibers lies in the ability to deliver biomolecules, for example, a core-shell nanofiber from co-axial electrospinning was previously employed for drug delivery [31,32]. 

Recently, surface characteristics have been enhanced by many surface modification methods, including the introduction of functional groups or using tools such as plasma treatment and laser ablation [12,33]. In addition, mineralization and crosslinking methods are modification steps used to improve the osteoconductive and mechanical properties necessary for bone tissue studies [34]. As shown in Figure 1, this review focuses on the development of electrospun fibers with modifications on cell-related studies for bone tissue engineering. In this review, we highlight the recent advances that upgraded electrospinning in many ways, including multi-axial electrospinning, patterned collectors, and melt-electrospinning. We summarize the modification approaches to alter the properties of electrospun materials to encourage cells to adhere, proliferate, and secrete tissue-specific matrices for bone tissue. Cell-related studies on bone tissues are reviewed due to the advanced use of electrospun nanofibers in preclinical studies. 

## 2. Nanofibrous Scaffold Fabrication

**Figure 2:**
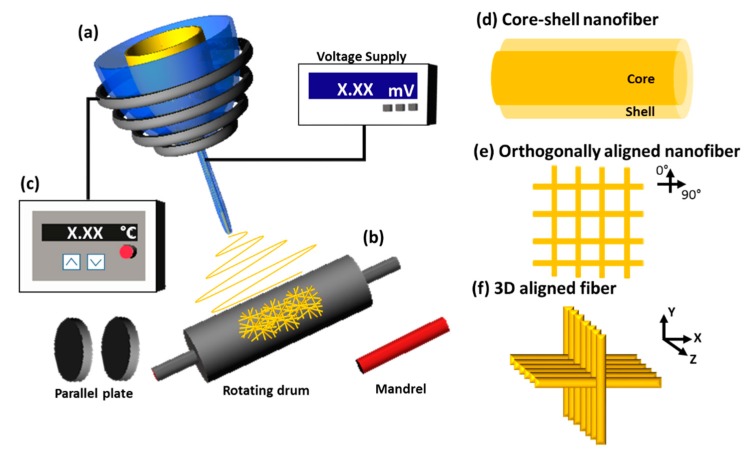
The development of electrospinning techniques according to the add-on tools. (**a**) Multi-axial spinneret, (**b**) developed collector, and (**c**) heating system for melt electrospinning. The illustration/photograph of apparatus and outcome fiber of each technique (**a**–**c**) is shown on the right side (**d**–**f**).

There are many ways to fabricate nanofibers, such as electrospinning, self-assembly, and phase separation [6,35,36]. Among these various techniques, the electrospinning method has gained attention due to its promising results for tissue engineering. Electrospinning has been developed to provide multiple components that mimic various aspects of tissue using multi-axial electrospinning [37]. The melt-electrospinning technique was invented to provide a 3D structure of the nanofiber [38]. The illustration of instrument set up and outcome fiber of each electrospinning technique was displayed in Figure 2. The detailed comparison of each electrospinning technique was described in Table 1. 

### 2.1. Conventional Electrospinning

The definition of electrospinning in principle is to apply high voltage (electro-) to a polymer solution, produce a stable jet of liquid, and transfer it to a solid fiber on the collector (-spinning). The process starts by applying electric potential to polymer solution, resulting in charge repulsion, which generates a force to overcome the surface tension of the polymer solution. Then, the stretching of the solution surface is performed, with a conical shape known as a Taylor core [44]. The stretching leads to the streaming of a jet of charged liquid that eventually solidifies, forming non-patterned nanofibers on the collector [45]. Even though this conventional method was introduced more than a decade ago, it is still used for bone tissue research. Chakraborty et al. optimized this technique by using a 90:10 (v/v) acetone-water solvent system to produce a highly porous regenerated cellulose nanofiber that supports cell proliferation and cell adhesion [39]. Nevertheless, this conventional method still has limitations, such as non-patterned orientation, lack of tensile strength, and a wide range of fiber thicknesses. Consequently, multi-axial electrospinning was further developed to allow the fabrication of composite nanofibers with a new structure and improved properties [8].

### 2.2. Multi-Axial Electrospinning (Core–Shell Nanofiber)

Hybrid and composite fibers can be made using two/multi-channel spinnerets/nozzles, allowing two or more solutions to be delivered into different channels simultaneously. Compared to conventional methods, multi-axial or co-axial electrospinning provides more spinnerets to produce core–shell nanofibers. The co-axial configuration is composed of two spinnerets: an inner-core spinneret surrounded concentrically by an outer-shell spinneret. When two polymer solutions are injected simultaneously, a core–shell droplet is produced at the exit of the inner and outer nozzles. The processes after that are similar to a conventional electrospinning, in which a collector is used to carry fibers. Co-axial electrospinning permits various materials, including polymers [46,47], oligomers [48], inorganic compounds [37], proteins [47], and biomolecules [41], to be immobilized into the core component of the core–shell nanofibers. Due to the functions of the core component, the core–shell nanofiber has more beneficial properties for bone tissue engineering. Recently, Shao et al. used a fibroin hybridized hydroxyapatite as the core material to improve mechanical properties. Tussah silk fibroin was used as a shell, fabricated by co-axial electrospinning using a green water solvent. The nanofiber effectively supported osteoblast-like MG-63 cell proliferation and promoted biomineralization [49]. Corresponding with Shao’s work, Tang et al. presented hydroxyapatite with poly(lactic-co-glycolic acid) PLGA as a nanofiber core, with high tensile strength, at 4.69 ± 0.51 MPa, for guided tissue regeneration (GTR) membranes [37]. The immobilization ability of the core–shell structure is also useful in drug delivery applications, which are important in bone tissue engineering. Gong et al. demonstrated drug delivery by nanofibers for bone regeneration. Poly(ethylene oxide) (PEO) containing bone morphogenetic protein 2 (BMP-2) was used as the inner core and poly-ε-caprolactone (PCL) was used as the outer shell [47]. Shalumon et al. fabricated silk fibroin (SF)/chitosan (CS)/nanohydroxyapatite (nHAP) nanofibers embedded with BMP-2 in the core of the nanofiber [50]. In addition, multi-layer of shell on core–shell nanofiber have been used for the controlled drug delivery. Different concentrations of the model active ingredient ketoprofen (KET) was combined with multiple layers of ethyl cellulose (EC) filament-forming matrix (outer, middle, and inner) for a zero-order drug delivery system [40]. Optimizing electrospinning conditions such as injection rate and applied electricity is important to prepare hollow, core–shell, or even triple-layer structure nanofibers. Triaxial fibers prepared by multi-axial electrospinning have an intermediate layer that can aid the delivery of multiple biomolecules in a multiple delivery system [17]. Because various materials can be used in triaxial fibers, the fiber characteristics such as the hydrophobicity and mechanical strength can be altered.

Multi-axial electrospinning not only provides the benefit of extra components but also prevents various problems encountered in electrospinning. A recent publication focused on the “lubrication effect” of the sheath solvent mixture, which has the ability to prevent clogging at the end of spinneret. Since the viscous polymer solution is surrounded by the solvent layer at the exit of the needle, improper drying and clinging problems due to the interfacial reaction between the polymer solution and the atmosphere can be prevented. Thus, monolithic fibers can be possibly made by the coaxial spinneret needles [51]. Core–shell structure nanofibers can also be made by three channel arrays of spinnerets. Yang et al. developed the modified tri-axial electrospinning method to prevent clogging in electrospinning [41]. Polyvinyl chloride (PVC) is selected as an antistatic coating material of spinnerets. The production of core–shell structures could be achieved by implementing a modified tri-axial process; in contrast, such structures are difficult to obtain from conventional co-axial electrospinning because a lubricating shell solvent is needed [41,51]. Increasing the number of spinnerets helps build up the core–shell structure, allowing the development of a drug delivery system for bone tissue engineering. However, the orientation of the nanofibers still needs improvement to mimic the natural cell environment.

### 2.3. Electrospinning with a Modified Collector (Oriented/Aligned Nanofiber)

The collector is an important component in electrospinning that allows for the preparation of oriented nanofibers by adjusting the speed of the collector. Many studies have increased the speed of the rotating collector [52,53,54,55,56,57,58]. When the collector is rotating, other parts of a conductive surface move closer to the end of the needle. The first electrostatic force produces the fiber on a conductive surface and then changes to the next surface, leading to the movement of fiber and creating fiber movement between surfaces. After that, the next electrostatic force is applied, stretching the fiber and enhancing alignment [56]. Hence, a high-speed rotation of the collector has been used to generate aligned nanofibers in many studies [52,53,54,55,56,57,58]. For example, aligned nanofibrous composites made of PLGA and PLGA/gelatin were fabricated and collected at a collector rotation speed of 2,000 rpm with a linear rate [52]. In another study, the orientation of cellulose nanofibers was enhanced by a high tangential speed of the rotating drum collector at 300 m/min [57]. Aligning the nanofiber helps direct the order of cell adhesion and leads to increased infiltration and cell viability. When aligned PLLA nanofibers were used with bone marrow stromal (BMS) cells, cell adhesion was enhanced [59]. Another study also used an aligned PLLA nanofiber with mesenchymal stem cells and found that the cell attachment and the ECM assembly in vitro are directed by the nanofiber morphology. The ECM protein collagen was assembled in order and single direction on the fiber, similar to the arrangement in a lamellar bone, resulting in anisotropic mechanical performance [53]. Cells orient along the direction of the nanofibers because of the maximal possibility of cells migrating in directions that are associated with the structural properties of fibers [60]. Oriented arrangement and elongation of cells could be guided by aligned electrospun PCL-PEG nanofibers. Moreover, cell infiltration and viability were enhanced, resulting in increased periodontal ligament-related gene expression [54]. Recent research also showed that the aligned PCL nanofibers combined with the surface-grafted bone forming peptide-1 improves osteogenesis of stem cells, even though it was performed in a non-osteoinductive environment [55]. Besides, the increase in the rotation speed resulted in thinner fibers due to the higher stretching level imposed on them [58]. This development allows for an orderly arrangement for nanofibers, which is important in scaffold design for bone tissue engineering.

Many studies have designed the collector to enhance fiber arrangement. Instead of rotating collector drums, aligned nanofibrous scaffolds can be obtained by using other kinds of collectors, including collecting mandrels [61], multiple plate collectors [62], and rotating wire drums [42]. Anindyajati et al. demonstrated enhanced orientation and mechanical properties of electrospun polycaprolactone upon using a mandrel-shaped collector with gaps. The structure of the resulting fiber exhibited multiple degrees of alignment and tensile properties that are potentially suitable for a fibrocartilage tissue engineering scaffold [56]. Another example of using a mandrel collector is electrospun P(LLA-CL) nanofiber yarn (yarn) collected by a rotating mandrel (60 rpm). A dynamic liquid-supporting system was used to fabricate the fiber for a subsequent freeze-drying step to become a scaffold for chondrogenic regeneration [61]. In addition, the direction of the nanofiber can be controlled by multiple parallel plates. In a study conducted by Mi et al., two parallel copper plates were used to fabricate unidirectionally arranged fibers and four orthogonal copper plates were used to fabricate orthogonally aligned fibers [62]. The wire drum is also popular for making aligned nanofibers. The aligned PCL nanofibers were collected at a distance of 13 cm from parallel copper wires as a collector and the orientation induced ordered mineralization, resulting in better mechanical properties than those of randomly oriented fibers [42].

### 2.4. Melt-Electrospinning (3-Dimensional Fiber)

The melt electrospinning method is an upgrade from the conventional electrospinning technique and involves the addition of a heat supply apparatus to create the fibers. The operation for melt electrospinning is similar to that for normal electrospinning, but the fiber transformation differs due to the heating process. The heating system melts the polymer, which then cools and solidifies instead of the solvent evaporating from the solution as in conventional electrospinning. Heat generated by a coil regulates the temperature of the gaps between the end of the needle and a collector. This technology is ecofriendly because it uses molten polymers as a substrate for spinning as a replacement for polymer solutions dissolved in organic solvents. Melt electrospinning can be potentially used in bone tissue engineering in cases where the issues of solvent recovery and toxicity are a concern [63]. Moreover, cytotoxicity is reduced in the melt-electrospinning technique because the fibers do not contain any residual harmful solvent. One excellent advantage that makes melt-electrospinning an outstanding candidate for bone tissue engineering is that it allows the creation of three-dimensional nanofibrous scaffolds [64]. Due to the diverse range of fiber diameters (from 270 nm up to 500 nm) that can be made by melt electrospinning, it is possible to fabricate three-dimensional structures that conventional processes cannot achieve. Due to this, large fiber pore sizes can be made by melt-electrospinning, leading to many advantages, including optimal cell invasion and growth, as well as vascular ingrowth for a highly vascularized tissue such as bone. Considerable research has been conducted on 3D structures involving the hybridization of nanofiber and microfiber scaffolds. For instance, melt-electrospinning combined with emulsion electrospinning approaches were used to produce PCL nanofibers and microfibers, which can deliver bFGF [65]. Due to the larger pore size in the 3D structure, they have a positive influence on cell migration, which is useful for the cell cascade. Kim et al. demonstrated the fabrication of nano/microfibrous composite scaffolds from silk fibroin and poly(ε-caprolactone). This hybrid method of solution electrospinning and melt electrospinning was used to fabricate a scaffold with a varying nanofiber composition [66].

Melt electrospinning can be further developed into melt-electrospinning writing (MEW), which can precisely control the macro structure and is also considered a class of 3D printing technology. The MEW process is largely based on near-field electrospinning combined with fused deposition modeling, in which a scaffold is designed from a computer software and produced using layer-by-layer deposition. Hence, 3D scaffolds and implants for bone tissue and vasculature can be created from the MEW technique. The electrospinning machine produces electrified molten jets that can directly write a 3D scaffold by a moving collector. A further advantage of the MEW process is the ability to control the balance of the pore size and pore interconnectivity [67]. A 3D poly(ε-caprolactone) scaffold was fabricated and its morphology was controllable for tissue engineering applications [38]. The diameter of these fibers can vary from 3 µm to 30 µm.

## 3. Modification/Post-Processing of Nanofiber for Bone Tissue Engineering

**Figure 3:**
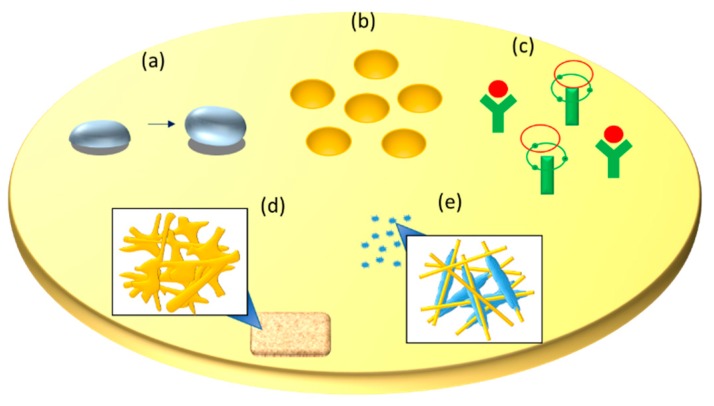
Properties after nanofiber modification. (**a**) increased hydrophilicity by plasma treatment, (**b**) macro-pore size structure using laser ablation, (**c**) chemically functionalized surface by wet chemical reaction, (**d**) increased compact structure by crosslinking process, and (**e**) use of hydroxyapatite as an osteoinductive agent by mineralization.

Producing a scaffold that can function like a native ECM is the common goal for bone tissue engineering, which can be achieved by further modification of nanofiber. The development of modification methods paves the way to fabricate scaffolds with controllable topography, pore characteristics, and surface properties. The currently used modification methods will be discussed in this section. Recently, the development of surface science has helped improve the surface characteristics of nanofibrous scaffolds by functionalization. Properties of a surface can be enhanced with various methods of surface modification as shown in Table 2, resulting in increased surface hydrophilicity and enhanced cellular behavior in tissue engineering, for example, high proliferation rate and bone formation. The illustration showing properties after each modification was shown in Figure 3.

### 3.1. Surface Modification: Plasma and Laser Treatment

Plasma treatment is a method for attaching polar groups on a surface, thereby improving the surface’s hydrophilicity and adhesion [73]. A proper choice of the plasma source allows various kinds of functional groups to be attached on the material exterior, resulting in high biocompatibility or the possibility of immobilizing bioactives such as ECM proteins, cationized gelatin [74], and RGD peptides [75]. Carboxyl groups or amine groups can be assembled by plasma treatments with ammonia, oxygen, or air, and they can improve cell proliferation and adhesion [68]. For example, PLGA nanofiber was treated with oxygen or ammonia plasma on its surface to improve its surface hydrophilicity, leading to cell adhesion and proliferation rate of fibroblast cells [8]. The purpose of surface modification, such as plasma treatment, is typically to improve biocompatibility. Sanders et al. demonstrated that plasma-induced surface polymerization was used to enhance the tissue compatibility of polyurethane fibrous scaffolds [76]. The negative and positive charges of monomers were introduced on the surface. The biocompatibility of the scaffold was investigated in the rat subcutaneous dorsum, and the surface was found to have negative charges supporting vessel growth in fibrous scaffolds [76]. Recently, plasma polymer deposition has been used to increase the density of functional groups on surfaces. Manakhov et al. demonstrated the reproducible deposition of amine and carboxyl plasma coating on PCL nanofibers via plasma polymer deposition, resulting in a high density of functional groups on the surface [68]. Besides plasma treatment, laser treatment is also a potential technique for enhancing fiber characteristics, especially porosity. The precision of the laser-generated machine provides treatment for specific tissues with reduced thermal damage and fewer impurities on the surface. Laser ablation or photo-ablation directly modifies the surface, and is advantageous as it is a non-contact and high precision process [77]. Fibers containing 3D microstructures can be made by ultra-short laser pulses (picosecond and femtosecond) with high precision. The designed patterns of scaffold surface structure from laser treatment provides the possibility to enhance cell adhesion, division, etc. [69].

### 3.2. Surface Functionalization 

Surface functionalization produces functional groups that can bind further with bioactive agents to enhance bone regeneration. Immobilizing biomolecules on the nanofiber surface via chemical modification is a potential method to deliver molecules since the immobilized compounds are attached on a surface by a covalent bond, which is difficult to break [78]. The EDC/NHS coupling method is commonly used for chemical conjugation. The mechanism of this coupling method is that first, EDC activates carboxyl groups and becomes an unstable intermediate. NHS is then added to stabilize that intermediate by changing it to an amine reactive sulfo-NHS ester, which can further form an amide bond with biomolecules [79]. Surface biomolecules can be ECM-derived proteins or growth factors or bioactive peptides [12,70,80,81]. Lee et al. demonstrated avidin surface functionalization on gelatin nanofibers using the EDC/NHS chemistry method. The avidin was used to bind with a biotin-FGF-2 complex to enhance cell proliferation [70]. Another study also showed that the ECM protein collagen can be functionalized onto the surface of nanofibers via an EDCEDC/HNS coupling method, and the scaffold can enhance osteogenic differentiation of mesenchymal stem cells [13]. Additionally, collagen immobilized nanofibers can be used in neural stem cell cultures, resulting in significantly enhanced cell adhesion and stem cell growth [82]. Besides EDC/NHS chemistry, a dopamine solution has been used recently for surface functionalization. The catechol groups in dopamine can be oxidized into quinone under a slightly alkaline condition and transform into polydopamine through an immobilization process [83]. Gao et al. demonstrated that BMP-7-derived peptides can be covalently immobilized onto the surface of polydopamine-coated PCL nanofibers [55]. Immobilizing these peptides on the nanofiber improved MSC adhesion and proliferation, which is useful for bone regeneration [55]. Moreover, surface functionalization by dopamine can be used as an intermediate to combine nanohydroxyapatite and PCL nanofibers for enhancing osteogenesis and biomineralization [83]. 

### 3.3. Inorganic Combination or Hydroxyapatite Deposition (Reinforced) Mineralization/Inorganic Hybrid

Nanofibers can be integrated with many kinds of inorganic materials to improve their mechanical functions; some can also be osteoinductive reagents. Natural bone is composed of hydroxyapatite (Ca_10_(PO_4_)_6_(OH)_2_; HA), which can be chemically synthesized and is commonly used with a bone tissue scaffold because of its biocompatibility and outstanding bioactivity in terms of its intrinsic osteoconduction and osteointegration potential [14,84]. However, HA can be brittle and may not be suitable to use as a component in the scaffold for bone tissue engineering. One solution to solve this issue is to hybridize HA with materials, such as polymers, that have ideal mechanical properties. Moreover, polymers offer enhanced biodegradability, biocompatibility, and cell adhesion. Thus, the combination of polymers and inorganic materials such as HA is common for enhancing bone tissue-related biological properties of the nanofibrous scaffold. Recently, Chahal et al. demonstrated the deposition of bone-like calcium phosphate onto electrospun hydroxyethyl cellulose upon incubating with simulated body fluid [71]. The composites such as bacterial cellulose (BCs)/HA exhibit enhanced osteoo-induction in stem cells [85,86]. PCL-HA composite nanofibers containing nano-HA particles also showed improved hMSC attachment, proliferation, and osteogenesis [14]. 

### 3.4. Crosslinking Methods

A potential scaffold for hard tissue engineering should provide controllable mechanical properties to support the tissue characteristics. Physical, enzymatic, and chemical crosslinking methods are potential methods to alter the mechanical properties of biomaterials. Among various kinds of cross-linkers, genipin (GP), a compound derived from gardenia extracts, has gained attention due to its biocompatibility. Commonly, GP can perform chemically crosslinking through amine groups between biological tissues and natural polymers [87]. It was demonstrated that the new bonds between fibers e.g. a silk fibroin–hydroxybutyl chitosan hybrid scaffold was formed through the binding of GP and free amine groups on the outside of the polymer chain [88]. The bonds increased the stiffness of scaffolds [88]. Ren demonstrated that, when genipin crosslinkers were incorporated into PCL/gelatin composite fibers, mechanical properties and osteogenesis capabilities were improved [87]. In addition, GP was used as a crosslinker on collagen nanofiber, and better cytocompatibility was observed compared to that upon use of a glutaraldehyde crosslinker [72]. Besides GP crosslinkers, citric acid has also been used as a non-toxic cross-linking agent. Citric acid was used to crosslink poly vinyl alcohol and was found to be non-cytotoxic [89]. Chen et al. fabricated PLA fiber with a citric acid crosslinker and found that the crosslinking agent also increased the apatite-nucleating capacity, inducing biomineralization in bone growth [90]. 

## 4. Conclusions

A considerable amount of literature is available that discusses recent research and considers the use of electrospun fiber scaffolds for bone tissue engineering applications. Since their structure mimics native tissue, nanofibrous scaffolds are promising biomaterials for use in bone tissue regeneration. With various techniques of fabrication already mentioned, polymeric nanofibrous scaffolds have the ability to be tailored and innovatively engineered depending on their use in bone tissue applications. The techniques used to achieve electrospun fibers are important and have been developed to design the structure, porosity, blending components, and size (diameter) of scaffolds. To enhance nanofibrous scaffolds after fabrication, surface modification, such as by wet chemistry (covalent) or mineralization, is performed in various ways to improve cell functions, such as proliferation and differentiation. The modified nanofibrous scaffolds have the potential to be successfully applied in clinical bone tissue regeneration. 

## Figures and Tables

**Table 1:** A comparison of the various processing methods for nanofibers.

Developments	Advantages	Limitations	Example of Recent Developments
Conventional electrospinning	- Facile and versatile method	- Non-patterned products - Lack of tensile strength	- Solvent system developed for high porosity fiber [39]
Multi-axial electrospinning	- Core-shell structure - Permits various materials to be immobilized, good for drug delivery - “Lubricant effect” prevents clog	- Toxic solvent - Poor cell infiltration	- Functional trilayer nanofibers for zero-order drug delivery [40] - Prevents jet instability by triaxial spinneret [41]
Electrospinning with a modified collector and high-speed rotation	- Aligned structure - Guides oriented arrangement and elongation of cells - Decrease in diameter - Good mechanical properties	- Toxic solvent - Complex setup - Clogging or jet instability can occur	- Hierarchically aligned polymer nanofiber as a bone scaffold [42]
Melt-electrospinning	- Three-dimensional structure - Larger pore size - Diverse diameter range - Eco-friendly method	- Cost for an extra instrument - Mostly amorphous fiber and thermal degradation	- Combination of nano- and micro-fibrous scaffolds for enhancing cell infiltration and bone tissue formation [43]

**Table 2:** A comparison of the various modification methods for nanofibers.

Developments	Advantages	Limitations	Example of Recent Developments
Plasma and laser treatment	- Improve surface hydrophilicity - Increase porosity - Increase cell adhesion and proliferation rate in fibroblast cells	- Fast degradation of functional groups on surface	- Plasma polymerization increases the density of functional groups [68] - Laser ablation on PCL/PVAc loaded hydroxyapatite [69]
Surface functionalization	- Strong bond, difficult to break - Diversity of functional groups - Provides delivery function	- Influencing the mechanical properties of the fiber - Batch-to-batch inconsistency	- Growth factor immobilization on gelatin nanofiber by avidin-biotin conjugation [70]
Inorganic combination	- Improve mechanical properties - Induces bone formation	- Compromising the porosity	- Bone-like calcium phosphate deposition onto cellulose fibers [71]
Cross-linking method	- Improved mechanical properties - Enhanced biodegradation time	- Cytotoxicity problem - Non-oriented structure	- Low-cytotoxicity crosslinking of nanofiber by the natural compound, genipin [72]
